# LongSAGE analysis of skeletal muscle at three prenatal stages in Tongcheng and Landrace pigs

**DOI:** 10.1186/gb-2007-8-6-r115

**Published:** 2007-06-16

**Authors:** Zhonglin Tang, Yong Li, Ping Wan, Xiaoping Li, Shuhong Zhao, Bang Liu, Bin Fan, Mengjin Zhu, Mei Yu, Kui Li

**Affiliations:** 1 Department of Gene and Cell Engineering, State Key Laboratory of Animal Nutrition, Institute of Animal Science, Chinese Academy of Agricultural Sciences, Beijing 100094, PR China; 2 Key Laboratory of Animal Genetics, Breeding and Reproduction of Ministry of Education of China, Huazhong Agricultural University, Wuhan 430070, PR China; 3 Shanghai Huaguan Biochip Co. Ltd, Shanghai, 201203, PR China; 4 Life and Environment Science College, Shanghai Normal University, Shanghai, 200234, PR China

## Abstract

**Background:**

Obese and lean pig breeds show obvious differences in muscle growth; however, the molecular mechanism underlying phenotype variation remains unknown. Prenatal muscle development programs postnatal performance. Here, we describe a genome-wide analysis of differences in prenatal skeletal muscle between Tongcheng (a typical indigenous Chinese breed) and Landrace (a leaner Western breed) pigs.

**Results:**

We generated transcriptome profiles of skeletal muscle from Tongcheng and Landrace pigs at 33, 65 and 90 days post coitus (dpc), using long serial analysis of gene expression (LongSAGE). We sequenced 317,115 LongSAGE tags and identified 1,400 and 1,201 differentially expressed transcripts during myogenesis in Tongcheng and Landrace pigs, respectively. From these, the Gene Ontology processes and expression patterns of these differentially expressed genes were constructed. Most of the genes showed different expression patterns in the two breeds. We also identified 532, 653 and 459 transcripts at 33, 65 and 90 dpc, respectively, that were differentially expressed between the two breeds. Growth factors, anti-apoptotic factors and genes involved in the regulation of protein synthesis were up-regulated in Landrace pigs. Finally, 12 differentially expressed genes were validated by quantitative PCR.

**Conclusion:**

Our data show that gene expression phenotypes differ significantly between the two breeds. In particular, a slower muscle growth rate and more complicated molecular changes were found in Tongcheng pigs, while genes responsible for increased cellular growth and myoblast survival were up-regulated in Landrace pigs. Our analyses will assist in the identification of candidate genes for meat production traits and elucidation of the development of prenatal skeletal muscle in mammals.

## Abstract 2

Transcriptional profiling of Tongcheng and Landrace pigs using long serial analysis of gene expression provides insight into the molecular mechanism underlying differences in muscle growth.

## Background

The pig (*Sus scrofa*) was domesticated over 7,000 years ago and has become one of the most important farm animals [1]. Anatomical, physiological, pathological and genomic similarities between pig and human have suggested that the pig could be considered a model species for human health issues [1-3]. Moreover, pigs have distinct advantages over other animals for studying the underlying mechanisms of phenotype variation within species: highly differentiated phenotypes resulting from intensive selection, and excellent phenotype records [4]. Therefore, use of pigs as research animals will benefit both animal agriculture and biomedical research.

Western pig breeds have been intensively selected over the past two decades for rapid, large and efficient accretion of muscle, which is believed to have led to deterioration in meat quality [5]. Landrace, a typical lean-type western breed, is now widely used for commercial production throughout the world. While indigenous Chinese pig breeds have lower growth rates and a lower lean meat content than conventional western pig breeds [6,7], they have proved superior in terms of perceived meat quality [8,9]. The Tongcheng variety is a typical indigenous Chinese breed of pig, and is one of the main groups derived from breeds in central China that have a coat color featuring two black ends. Tongcheng was also listed as an important breed for resource conservation by the Chinese Ministry of Agriculture in 2000.

In the pig, genotype has a major effect on embryonic growth rate [10]. Preimplantation embryos from Meishan (an indigenous Chinese breed) females have markedly slower growth rates through day 12 than embryos from Yorkshire (a western breed) females [10-12]. However, there are no current reports of the differences between indigenous Chinese and western pigs in prenatal skeletal muscle development. The lower potential for postnatal muscle growth in indigenous Chinese breeds compared with exotic breeds is already evident at birth in the lower total number of fibers (TNF), which is fixed before birth [13,14]. Hence, prenatal skeletal muscle development is an important determinant of both muscle growth and meat quality [15]. Myogenesis is a highly ordered process that can be subdivided into a sequence of temporally separable events: myogenic progenitor cell determination and proliferation, myoblast differentiation, and subsequent myotube modulation. Establishment of the TNF involves two major waves of fiber generation: a primary generation from 35 to about 60 days post coitus (dpc), and a secondary generation from about 54 to 90 dpc [13]. Hence, around 35 dpc, 60 dpc and 90 dpc are key time points in prenatal skeletal muscle development. More systematic analyses of these particular stages are required to elucidate these phenomena further.

Comparative analyses of expression profiles are useful for identifying the molecular differences between variant muscle phenotypes [16]. Full-transcriptome analysis of skeletal muscle may be particularly valuable for such studies. In recent years, several techniques have been used to elucidate the molecular basis of prenatal skeletal muscle development [17-19]. However, the genetic complexity underlying the development of skeletal muscle remains only partially understood. In particular, there have been no reports on the differences in the global transcription profiles of prenatal skeletal muscle between indigenous Chinese and western breeds of pig. Consequently, a genome-wide profiling of transcription is needed as a basis for further understanding of the molecular basis of prenatal skeletal muscle development by analyzing gene expression patterns of prenatal skeletal muscle development at key stages and assembling molecular mechanisms. This would also help to identify putative candidate genes for meat production traits. The analysis of gene expression will also facilitate the study of gene function.

Serial analysis of gene expression (SAGE) is a powerful tool for the comprehensive and quantitative measurement of gene expression and for identifying novel genes [20,21]. In addition, the results from experiments undertaken in different laboratories can be compared [22]. Long serial analysis of gene expression (LongSAGE) has a higher specificity for gene identification than conventional SAGE [23]. In this study, LongSAGE was used to investigate the molecular basis of the differences in postnatal development between indigenous Chinese and western breeds by analyzing and comparing prenatal muscle gene expression in Tongcheng and Landrace pigs. We describe the construction and screening of six LongSAGE libraries constructed from Tongcheng (T) and Landrace (L) pigs at 33, 65, 90 dpc, designated T33, T65, T90, L33, L65 and L90. To delineate the genes that were differentially expressed at these three developmental stages and also between breeds, the LongSAGE libraries were further subjected to pairwise comparisons. Through Gene Ontology (GO) annotation and cluster analyses for these differentially expressed transcripts, we have obtained the first results showing the gene regulation patterns during prenatal skeletal muscle development in these two breeds of pig.

## Results

### LongSAGE libraries

**Figure 1:**
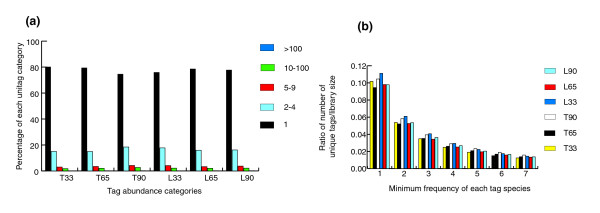
Genetic complexity of prenatal skeletal muscle of pigs. **(a) **Distribution of LongSAGE tags in abundance categories. The number of unique transcripts (tags) for each abundance category is shown. T, Tongcheng; L, Landrace; 33, 65 and 90 refer to days post coitus; 1, 2-4, 5-9, 10-100 and >100 indicate tag abundance categories in our LongSAGE libraries. **(b) **Genetic complexity of the Tongcheng pigs in comparison with the Landrace variety during skeletal muscle development.

A combined total of 317,115 LongSAGE tags were sequenced from the six LongSAGE libraries. This translated into 98,437 distinct transcripts. Approximately 75% to 80% (83,754) of these unique tags were observed only once in each library (Figure 1a). All the libraries were very similar in the total number of tags identified (approximately 50,000 per library), as well as average GC content (44.56% to 50.02%) (Table 1; also deposited in the NCBI database (GSM125246, GSM125247, GSM125248, GSM125249, GSM125250, and GSM125251)). Moreover, the ratio of unique tags to total tags was reduced in parallel with the development of skeletal muscle for Tongcheng pigs (Table 1). This suggested that more genes were detected at early stages than at later stages in Tongcheng pigs. Also, more transcripts were expressed at lower levels during early stages of skeletal muscle development in this breed. However, we observed the opposite change in Landrace pigs (Figure 1b). These results suggest that more intricate molecular events occur during early stages of skeletal muscle development in Tongcheng pigs, but during later stages in Landrace pigs.

**Table 1:** Summary of data obtained from the LongSAGE libraries

	LongSAGE library					
	
	T33	T65	T90	L33	L65	L90
Total tags^1^	50,450	53,927	53,761	53,104	54,483	51,188
Unique tags^2^	25,738	24,655	22,035	24,408	24,829	22,464
Unique tags/total tags (%)^3^	51.0	45.7	41.0	46.0	45.6	43.9
Tags/clone	23.4	26.5	31.7	25.97	24.8	25.9
Average GC content^4^	45.16	50.02	44.56	47.24	47.15	46.20
Remaining total tags^5^	32,722 (64.8)	37,058 (68.6)	40,074 (74.5)	37,135 (69.8)	37,948 (69.6)	36,596 (71.4)
Remaining unique tags^6^	8,081	7,786	8,348	8,439	8,294	7,872
Unmatched tags^7^	2,458 (30.4%)	2,377 (30.5%)	2,621 (31.4%)	2,362 (28.0%)	2,740 (33.0%)	2,513 (31.9%)
Matched tags^8^	5,623 (69.6%)	5,409 (69.5%)	5,727 (68.6%)	6,077 (72.0%)	5,554 (67%)	5,359 (68.1%)
Single match^9^	5,437 (96.7%)	5,249 (96.9%)	5,550 (97%)	5,887 (97%)	5,358 (96.8%)	5,163 (96.7%)
Multiple matches^10^	188 (3.3%)	169 (3.1%)	174 (3.0%)	185 (3.0%)	175 (3.2%)	176 (3.3%)

^1^Total tags were obtained in each library. LongSAGE tags containing wildcard characters not in {A, C, G, T} were discarded. ^2^Unique tags were obtained in each library. ^3^The differences in accrual rates (the ratio of unique tags to total tags) indicate that the number of genes expressed regularly changed during myogenesis. ^4^For an explanation of this value, in the context of the quality of a SAGE library, see Margulies *et al*. [31]. ^5^The total tags remaining in each library after eliminating the unique tags with a frequency <2 in all six libraries. The percentage of non-singleton tags is shown in parentheses. ^6^The unique tags remaining in each library after eliminating the unique tags with a frequency <2 in all six libraries. ^7^Unique tags unmatched with any known sequence. The values in parentheses indicate the percentage of unique tags in the total. ^8^Unique tags that correspond to UniGene entries. The values in parentheses indicate the percentage of unique tags in the total. ^9^Unique tags matched with a single UniGene sequence. ^10^Unique tags matched with more than one UniGene sequence. T, Tongcheng; L, Landrace; 33, 65 and 90 refer to days post coitus.

A total of 83,754 unique tags, which were not observed more than twice in any of the six libraries, were eliminated from the analysis to compensate for possible sequencing errors [24]. The remaining 14,683 valid unique tags were then selected for further comparative analysis. As shown in Table 1, the percentage of unique tags assigned to UniGene entries ranged from 67% to 72%. Of these, about 97% corresponded to single UniGene entries, whereas approximately 3% matched more than one UniGene cluster because they contained a 3' region conserved between different genes. In addition, these unique tags matched at the punctuation mark (CATG) in all the UniGene clusters. A total of 5,953 unique tags were unmatched by any known sequence in the combined LongSAGE libraries, while the occurrence of unknown tags was probably due to the incompleteness of pig genome sequencing [2,25].

### Validation of LongSAGE data by quantitative PCR

To confirm that the genes identified were differentially expressed, we selected 12 genes for validation by quantitative PCR (QPCR) on the basis of their functional roles in skeletal muscle development and expression patterns in these libraries. Among these genes, five encoding myofibrillar proteins (fast skeletal myosin light chain 2 (*MYLPF*); myosin, light chain 2, regulatory, cardiac, slow (*MYL2*); myosin, light chain 1, alkali, skeletal, fast (*MYL1*); sarcolipin (*SLN*); and troponin C type 2, fast, (*TNNC2*)), and two encoding proteins involved in regulation of myoblast proliferation and differentiation (lectin, galactoside-binding, soluble, 1 (galectin 1; *LGALS1*); and transducer of ERBB2, 1 (*TOB1*)) were selected for validation. Three genes, RPS28 (ribosomal protein S28), *GNB2L1 *(guanine nucleotide binding protein (G protein), beta polypeptide 2-like 1), and *TPT1 *(tumor protein, translationally controlled 1), which are associated with protein synthesis, were selected because their expression levels differed significantly between the two breeds at 65 dpc. Validation was also performed for the cellular retinoic acid binding protein 1 (*CRABP1*) gene, which was expressed specifically at 33 dpc in both breeds. Finally, a noncoding RNA, named trophoblast-derived noncoding RNA (*TncRNA*), which was up-regulated during myogenesis in both breeds, was identified and selected for validation by QPCR. Housekeeping genes such as those encoding β-actin (*ACTB*) and glyceraldehyde-3-phosphate dehydrogenase (*GAPDH*), commonly used as internal controls for such analysis, were not suitable for normalization in these experiments because their transcription was altered during myogenesis [18,26]. Histone 3 mRNA (H3 histone, family 3A (*H3F3A*)), which was consistently expressed in our study, was therefore used as an internal control. The results for a panel of the 12 genes were in good agreement with the LongSAGE data (Table 2) and there was a highly significant correlation (r = 0.79, p = 8.52E-17) between the two techniques. For example, genes encoding myofibrillar proteins, such as *MYL1*, *SLN*, *MYLPF *and *TNNC2*, were shown to be up-regulated during myogenesis in both the LongSAGE and QPCR experiments, while QPCR also showed a significant difference between the two breeds in the expression of *GNB2L1 *and *TPT1 *at 65 dpc. For *CRABP1*, although LongSAGE tags were not detected in skeletal muscle from either breed at 65 or 90 dpc, QPCR indicated that it was expressed at low levels. This correlation indicated that our LongSAGE results reliably reveal the differences in gene expression profiles in skeletal muscle.

**Table 2:** Genes differentially expressed in LongSAGE data and validated by QPCR

Gene	Method*	Fold changes of gene expression in different skeletal muscle samples					
		
		T33	T65	T90	L33	L65	L90
*MYLPF*	QPCR	1.00^a^	8.00^b^	16.00^c^	0.71^a^	10.56^b^	39.40^d^
	SAGE	1.00	4.10	4.63	0.67	2.31	2.34
*MYL2*	QPCR	1.00^a^	4.92^b^	2.46^ab^	0.31^c^	4.59^b^	2.64^b^
	SAGE	1.00	1.41	2.56	0.23	2.70	2.23
*MYL1*	QPCR	1.00^a^	9.85^b^	13.00^c^	0.41^a^	12.13^bc^	34.30^d^
	SAGE	1.00	6.62	11.85	0.38	6.31	5.77
*SLN*	QPCR	1.00^a^	40.50^b^	78.79^c^	0.71^a^	64.00^b^	207.94^d^
	SAGE	1.00	16.70	29.30	0.20	14.70	24.00
*TNNC2*	QPCR	1.00^a^	22.63^b^	6.50^c^	0.27^d^	6.96^c^	8.57^c^
	SAGE	1.00	6.74	5.52	0.26	4.03	3.58
*TOB1*	QPCR	1.00^a^	2.30^a^	7.46^b^	1.53^a^	3.73^c^	1.87^a^
	SAGE	1.00	3.00	11.00	1.00	3.00	2.00
*CRABP1*	QPCR	1.00^a^	0.07^b^	0.04^b^	2.64^c^	0.05^b^	0.05^b^
	SAGE	1.00	0.00	0.00	3.36	0.00	0.00
*LGALS1*	QPCR	1.00^a^	0.57^b^	0.62^bc^	0.41^b^	0.74^b^	0.38^b^
	SAGE	1.00	0.86	0.86	0.88	1.26	0.80
*GNB2L1*	QPCR	1.00^a^	0.01^b^	0.76^a^	0.76^a^	1.00^a^	0.54^a^
	SAGE	1.00	0.30	1.02	0.93	1.02	0.80
*TPT1*	QPCR	1.00^a^	0.00^b^	2.00^c^	1.07^a^	2.83^c^	1.74^ac^
	SAGE	1.00	0.74	2.69	0.93	1.61	1.41
*RPS28*	QPCR	1.00^a^	0.81^ab^	1.00^a^	0.62^b^	0.81^ab^	0.81^ab^
	SAGE	1.00	0.39	1.02	0.81	1.06	1.06
*TncRNA*	QPCR	1.00^a^	0.81^a^	5.66^b^	0.27^c^	1.00^a^	1.62^d^
	SAGE	1.00	1.50	29.50	0.50	1.00	5.00

*The QPCR row provides the ratio of the 2^-ΔΔCt ^value for each breed/stage sample to the 2^-ΔΔCt ^of T33. For the T33 sample, the fold change in gene expression relative to the T33 equals one, by definition. For each row, different superscript letters (a, b or c) indicate a statistically significant difference in gene expression between skeletal muscles at *p *< 0.05 in the QPCR experiment; the same superscript letters indicate no statistically significant difference (*p *> 0.05). For values with two superscript letters, gene expression in that sample was not significantly different from the expression in the other two samples compared, but the other two samples were significantly different in gene expression; for example, for the *MYL2 *gene, the value 2.46^ab ^indicates that the expression in T90 was not statistically significantly different from that of both T33 and T65, but there was a significant difference between the expression of T33 (1.00^a^) and T65 (4.92^b^). The SAGE row provides the frequency of each breeds/stage sample relative to T33 frequency in the LongSAGE data. T, Tongcheng; L, Landrace; 33, 65 and 90 refer to days post coitus.

### Cluster analysis

**Figure 2:**
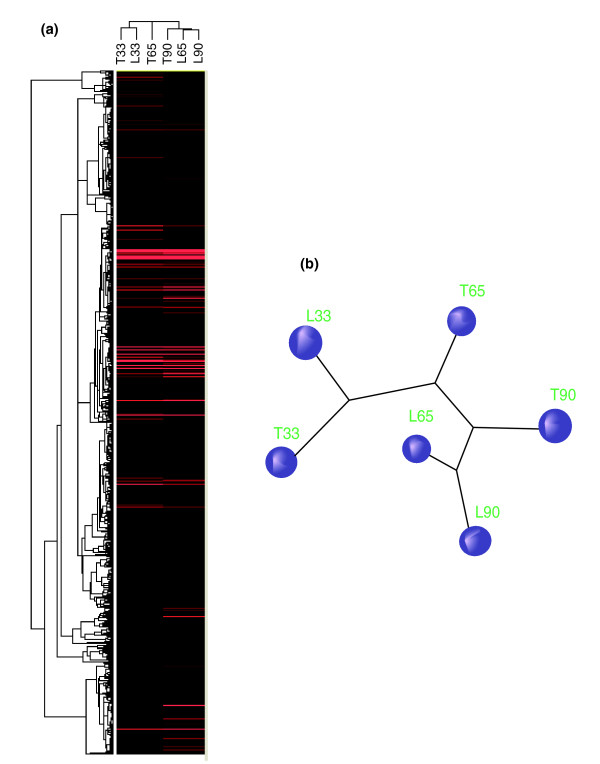
Similarity of transcriptome profiles between six muscle tissues using cluster analysis. **(a) **Clustering dendrogram of LongSAGE libraries generated by Cluster 3.0 and TreeView software. **(b) **Hypothetical tree-like diagram generated by TreeBuild 3.0 software, indicating the relatedness of these six libraries.

To gain insight into transcriptome-scale similarities among all six skeletal muscle libraries, we performed systematic cluster analysis using two different methods (Cluster 3.0 and TreeBuild 3D software) independently. Both sets of results indicated that the six different transcription profiles could be divided into three distinct classes (Figure 2). L65 and L90 were initially clustered together because their expression profiles were most similar, and T90 was then grouped into this class by similarity to both of them. T33 and L33 were clustered to form another class. Interestingly, T65 differed from the other five samples in transcriptional profiling and was clustered into a single class. Also, the gene expression patterns in Landrace pigs at 65 and 90 dpc were more similar than those in Tongcheng pigs.

### Comparisons of the gene expression profiles between Landrace and Tongcheng pigs during skeletal muscle development

**Figure 3:**
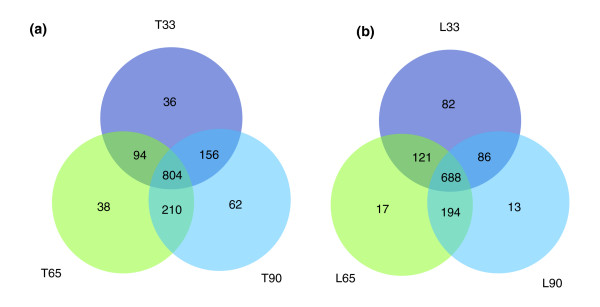
Venn diagrams of genes differentially expressed at different stages. There were 1,400 and 1,201 differentially expressed tags for **(a) **Tongcheng and **(b) **Landrace pigs, respectively. This figure is not drawn to scale. T, Tongcheng; L, Landrace; 33, 65 and 90 refer to days post coitus.

Table 3 shows the comparison of differentially expressed tags between the libraries. A total of 1,400 and 1,201 unique tags were differentially expressed during skeletal muscle development in Tongcheng and Landrace pigs, respectively. Among these tags, 234 (corresponding to 182 annotated transcripts) and 203 (corresponding to 153 annotated transcripts) matched annotated genes in the Tongcheng and Landrace breeds, respectively. Figure 3 shows the distribution of differentially expressed tags at each stage. It reveals that most of these transcripts were expressed in all the skeletal muscle samples at each of the three selected stages. Only a few were restricted in regulation of expression to a single stage.

**Table 3:** Number of differentially expressed genes and node distance between six skeletal muscle samples

	T33	T65	T90	L33	L65
T65	701 (2.24)				
T90	751 (1.96)	781 (1.99)			
L33	532 (1.38)	988 (2.51)	1,008 (2.42)		
L65	645 (1.51)	653 (1.52)	577 (1.43)	741 (1.76)	
L90	684 (1.83)	697 (1.76)	459 (1.3)	812 (2.11)	341 (1.26)

The values in parentheses indicate the node distance between skeletal muscles in cluster analysis using TreeBuild 3.0. T, Tongcheng; L, Landrace; 33, 65 and 90 refer to days post coitus.

#### Gene Ontology analysis

**Figure 4:**
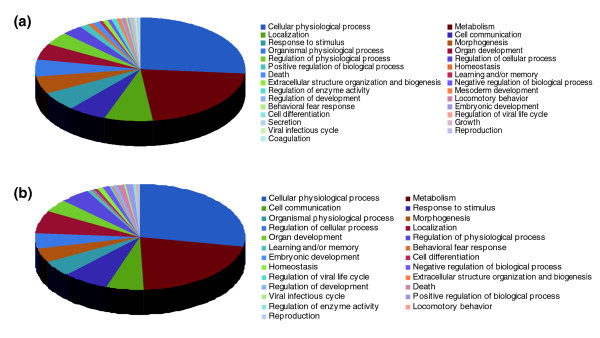
GO classifications of biological processes of genes differentially expressed during skeletal muscle development. On the basis of the annotated genes that matched our unique tags, GO analysis was carried out using the Blast2GO program [66]. The numbers shown indicate the exact number of genes for each GO classification. **(a) **GO categories for Tongcheng pigs. **(b) **GO categories for Landrace pigs.

To gain further insight into the biological importance of the differentially expressed transcripts identified, we further analyzed the functional categories of the annotated genes by querying their associated Gene Ontologies. In general, the categories of biological processes involved in myogenesis were similar in Tongcheng and Landrace pigs. Mainly, they included cellular physiological pathways, metabolism, localization processes, cell communication, responses to stimuli and development (Figure 4) (at level 3). However, the numbers of differentially expressed genes involved in certain biological processes (at level 5) were quite different in Tongcheng and Landrace pigs. For instance, more genes involved in cellular biosynthesis (T versus L = 21.32% versus 9.77%, *p *= 0.00646), regulation of cell proliferation (T versus L = 3.55% versus 0%, *p *= 0.04446), organic acid metabolism (T versus L = 6.70% versus 0.79%, *p *= 0.07322), macromolecule biosynthesis (T versus L = 14.21% versus 7.52%, *p *= 0.07818), and regulation of cell size (T versus L = 3.05% versus 0%, *p *= 0.08482) were differentially expressed in Tongcheng pigs. In contrast, there was a tendency for more differentially expressed genes involved in biopolymer metabolism (L versus T = 30.83% versus 22.34%, *p *= 0.09562) to be identified in Landrace pigs.

#### Expression patterns

In order to determine whether the temporal pattern of expression of a gene during prenatal skeletal muscle development might predict its molecular function, clusters of differential expression tags were assembled. The differentially expressed genes identified in our screening were found to exhibit eight types of pattern in both Tongcheng and Landrace pigs (Additional data files 1 and 2 list all the LongSAGE tags used in this analysis and their corresponding cluster assignments for Tongcheng and Landrace pigs, respectively). These patterns are shown graphically for each breed in Additional data file 6. Table 4 lists the genes that had previously been confirmed (Additional data file 7) to be either highly or specifically expressed in developing skeletal muscle and for which the specific GO category assignments were enriched in each expression pattern cluster for both pig breeds.

**Table 4:** Summary of LongSAGE tag cluster data according to breed type

Cluster*	Tags per cluster^†^	Previously characterized genes^‡^	GO categories enriched in the cluster^§^
**Tongcheng**			
1	191	*APOBEC2*, *BIN1*, *BTBD1*, *CACNB1*, *MYBPC1*, *MYL1*, *MYOZ1*, *RRAS*, *RTN4*, *SH3BGR*, *SLN*, *TCAP*, *TMOD1*, *TNNC2*, *TNNT3*, *TPM1*	Muscle development (0.0007806)
2	245	*ACTN2*, *AMPD1*, *ATP2A1*, *CKM*, *DES*, *ENO3*, *MYH2*, *RYR1*, *TMOD4*, *TPM2*, *GAPDH*, *IGF2*	Carbohydrate metabolism (0.001492) Mitochondrion (0.016297)
3	204	*LASS2*	Signal transduction (0.008368)
4	160	*SPARC*, *HSPB2*, *ATF4*, *MYL2*	Tricarboxylic acid cycle (0.021523489)
5	152	*MYH3*, *S100A11*	Ribosomal (1.25E-06)
6	77		
7	218	*PSMD2*, *RTN2*, *SERF1A*, *THBS4*, *TIMM13*, *TNNC1*, *TNNI1*, *TNNT1*, *FAU*, *HSPA8*, *FHL1C*, *HDAC5*, *MYOT*	Obsolete molecular function (0.048234)
8	153	*DGKZ*, *GNB2L1*	Binding (0.023903)
Total	1,400		
**Landrace**			
1	158	*APOBEC2*, *CFL2*, *HSPB2*, *ITGB1*, *MYH3*, *PGM5*, *RTN4*, *TNNT1*, *TPM2*, *FHL1C*	Muscle development (0.003181)
2	94	*SPARC*, *TIMM13*	Mitochondrion (0.002539)
3	126	*AMPD1*, *CKM*, *DES*, *MYBPC1*, *MYL1*, *MYOZ1*, *PFDN5*, *RYR1*, *SLN*, *TNNT3*, *MYL2*	Muscle contraction (0.035242)
4	160	*LGALS1*, *SERF1A1*, *FAU*, *HSPA8*	Ribosomal (4.83E-07)
5	84		Cytoskeleton organization and biogenesis (0.012664)
6	301	*GSN*, *TPM3*, *TPM4*, *TRIO*, *S100A11*, *NACA*, *SUMO2*	Cell cycle (0.002587)
7	95	*CACNB1*, *MYLPF*, *TMOD1*, *TNNI1*, *IGF2*	Development (0.014224)
8	183	*ACTN2*, *ATP2A1*, *BIN1*, *CCNG1*, *ENO3*, *MYH2*, *RTN2*, *SH3BGR*, *TCAP*, *TPM1*, *GAPDH*, *SDHD*, *HDAC5*	Protein complex assembly (0.035886)
Total	1,201		

*The expression patterns in each cluster for Tongcheng and Landrace pigs are shown as (A) and (B) in Additional data file 6, respectively. ^†^Tags in cluster value corresponds to the number of LongSAGE tags in each cluster. ^‡^Selected genes previously detected during myogenesis are indicated (references for these are given in Additional data file 7). ^§^All significant (*p *< 0.05) GO categories over-represented in individual clusters were determined by EASE scores [68]; raw EASE scores (Jackknife one-sided Fisher exact *p *values) for the categories in question are given in parentheses.

Most of the genes previously reported to be regulated in porcine prenatal skeletal muscle were detected in our analysis and shared similar expression patterns [17,18]. For instance, expression of desmin (*DES*) and *GAPDH *was increased during myogenesis in both breeds, but both vimentin (*VIM*) and eukaryotic translation elongation factor 1 alpha 1 (*EEF1A1*) showed lower expression levels. These data are consistent with previous reports [17,18]. Some genes that have been shown to play important roles in the development of skeletal muscle in humans and model animals [27,28], but had not been identified in pig, were also detected in our analysis. These included *SUMO2 *(SMT3 suppressor of mif two 3 homolog 2 (*Saccharomyces cerevisiae*) and *LGALS1*, which have essential functions during myotube formation [27,28]. *SUMO2*, a member of the SUMO gene family, and *LGALS1 *were the only differentially expressed genes of this type found in Landrace pigs.

Certain functional categories of genes were over-represented in a number of LongSAGE tag clusters (Table 4). In Tongcheng pigs, muscle development genes, which are typically up-regulated in development, were enriched in cluster 1. Cluster 2 was enriched in mitochondrial proteins and carbohydrate metabolism. Tricarboxylic acid cycle genes were concentrated in cluster 4. Ribosomal proteins, which showed lower expression in the later stages of development, were highly enriched in cluster 5. Genes representing a number of other functional categories were also enriched in specific clusters; for example, genes involved in signal transduction, obsolete molecular function and protein binding in clusters 3, 7 and 8, respectively. In Landrace pigs, by contrast, muscle development and muscle contraction genes were enriched in clusters 1 and 3, respectively. Mitochondrial proteins were concentrated in cluster 2. Ribosomal proteins were obviously enriched in cluster 4. In addition, genes involved in cytoskeleton organization and biogenesis, cell cycle and protein complex assembly, which were concentrated in clusters 5, 6 and 8, respectively, were not enriched in the Tongcheng clusters. On the other hand, genes for signal transduction, the tricarboxylic acid cycle and obsolete molecular function were not over-represented in Landrace pigs.

### Differential expression of genes between Tongcheng and Landrace pigs at the same stage of skeletal muscle development

#### T33 versus L33

**Figure 5:**
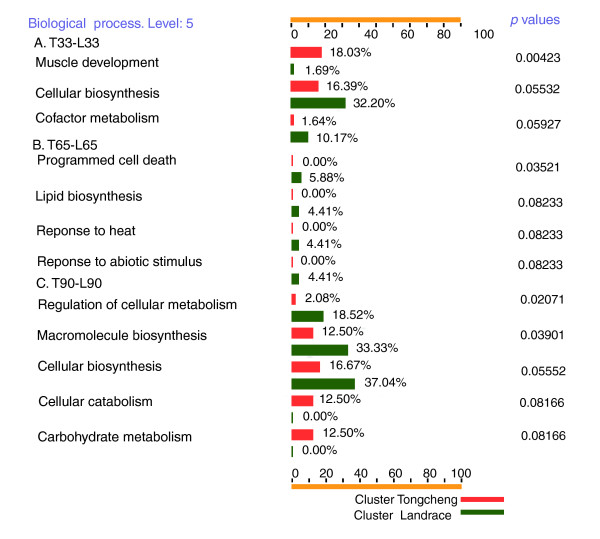
GO annotations for 'biological process' for differentially expressed genes between breeds at specific stages. These categories include only Gene Ontologies with significant difference in gene numbers between breeds (*p *< 0.10). Numbers of up-regulated genes in Tongcheng pigs were compared with those in Landrace pigs by the FatiGO tool and *p *values <0.10 were considered significant. Gene Ontologies are listed on the vertical axis. The score on the horizontal axis is the percentage of up-regulated genes. T, Tongcheng; L, Landrace; 33, 65 and 90 refer to days post coitus.

We identified 532 tags that were differentially expressed between the T33 and L33 samples, including 327 known genes or expressed sequence tags (ESTs) and 105 novel tags. Among these genes, 221 were expressed more abundantly in T33, while 311 were expressed at higher levels in L33. Analysis of the GO annotations indicates that more genes encoding proteins associated with muscle development (18.03% versus 1.69% for T33 versus L33, *p *= 0.00423) were up-regulated in Tongcheng pigs, whereas more genes related to cellular biosynthesis (16.39% versus 32.20% for T33 versus L33, *p *= 0.05927) and cofactor metabolism (1.64% versus 10.17% for T33 versus L33, *p *= 0.05532) were up-regulated in Landrace pigs (Figure 5a). We further focused on 67 transcripts that showed significant fold differences ≥2.0 (*p *< 0.01) and tag counts ≥10 in any of our SAGE libraries (Additional data file 3). Among these genes, the following were more highly expressed in T33: *PDLIM7 *(PDZ and LIM domain 7 (enigma)), *CAPNS1 *(calpain, small subunit 1), *ACTC *(actin, alpha, cardiac muscle), *TNNC2*, *FSCN1 *(fascin homolog 1, actin-bundling protein (*Strongylocentrotus purpuratus*)), *COL1A1 *(collagen, type I, alpha 1), *MYL2*, *ACTG1 *(actin, gamma 1), and *MYH3 *(myosin, heavy polypeptide 3, skeletal muscle, embryonic). It is obvious that most of these genes are related to muscle fiber formation. In contrast, the following were more highly expressed in L33: *MARCKS *(myristoylated alanine-rich protein kinase C substrate), *TSC22D1 *(TSC22 domain family, member 1), *CRABP1*, *PTMA *(prothymosin, alpha (gene sequence 28)), *GSTP1 *(glutathione S-transferase pi), *FAU *(Finkel-Biskis-Reilly murine sarcoma virus (FBR-MuSV) ubiquitously expressed (fox derived)), *UCHL1 *(ubiquitin carboxyl-terminal esterase L1 (ubiquitin thiolesterase)), *MDK *(midkine (neurite growth-promoting factor 2)), and *GNAS *(GNAS complex locus). Interestingly, we also detected several genes in one breed only. For example, *DNAJC5 *(DnaJ (Hsp40) homolog, subfamily C, member 5) and *RPL9 *(ribosomal protein L9) were not detectable in L33, whereas *RPL29 *(ribosomal protein L29), *PSMB2 *(proteasome (prosome, macropain) subunit, beta type, 2), *RPS4X *(ribosomal protein S4), and *SLC25A6 *(solute carrier family 25 (mitochondrial carrier; adenine nucleotide translocator), member 6) were absent from T33.

#### T65 versus L65

A total of 653 transcripts were differentially expressed between T65 and L65, including 497 annotated genes or EST sequences and 156 novel tags. Of these, 342 were up-regulated in T65 and 311 were more highly expressed in L65. Analysis of the biological processes associated with these factors suggests that more genes related to programmed cell death (0% versus 5.88% for T65 versus L65, *p *= 0.03521), lipid biosynthesis (0% versus 4.41% for T65 versus L65, *p *= 0.08233), response to heat (0% versus 4.41% for T65 versus L65, *p *= 0.08233) and responses to abiotic stimuli (0% versus 4.41% for T65 versus L65, *p *= 0.08233) were up-regulated in Landrace pigs (Figure 5b). One hundred and nineteen unique tags were differentially expressed with ≥2.0-fold difference (*p *< 0.01) between the two breeds at 65 dpc (Additional data file 4). Among these transcripts, ribosome families were the most variable. Most of these genes were more highly expressed in Landrace pigs, for example, those encoding ribosomal proteins L36 (*RPL36*), L38(*RPL38*), S26 (*RPS26*) and S28 (*RPS28*). The following were also more highly expressed in L65: *IGF2 *(insulin-like growth factor 2 (somatomedin A)), *GNB2L1*, *DES *(desmin), *ALDOA *(aldolase A, fructose-bisphosphatase),*CD63 *(CD63 molecule), *TTN *(titin), *TPT1*, and *RYR1 *(ryanodine receptor 1). On the other hand, the following were more highly expressed in T65: *Cox6c *(cytochrome c oxidase subunit Vic), *FAU*, *PCBP4 *(poly(rC) binding protein 4), *PPP1R14B *(protein phosphatase 1, regulatory (inhibitor) subunit 14B), *FHL1C *(four and a half LIM domains 1 protein, isoform C), *THBS4 *(thrombospondin 4), *TMOD1 *(tropomodulin 1), and *YWHAQ *(tyrosine 3-monooxygenase/tryptophan 5-monooxygenase activation protein, theta polypeptide). Interestingly, four genes were found to be absent from T65: *VCP *(valosin-containing protein), *RPL29*, *SULT1E1 *(sulfotransferase family 1E, estrogen-preferring, member 1), and *RPLP0 *(ribosomal protein, large, P0). Moreover, six transcripts were detectable in T65 only, including *PRDX3 *(peroxiredoxin 3), *BCAP31 *(B-cell receptor-associated protein 31) and *TH1L *(TH1-like).

#### T90 versus L90

We found that 459 transcripts, including 330 annotated genes and ESTs and 129 novel tags, were differentially expressed between T90 and L90. Of these transcripts, 273 were up-regulated in T90. More genes related to the regulation of cellular metabolism (2.08% versus 18.52% for T90 versus L90, *p *= 0.02071), macromolecule biosynthesis (12.50% versus 33.33% for T90 versus L90, *p *= 0.03901), and cellular biosynthesis (16.67% versus 37.04% for T90 versus L90, *p *= 0.05552) were up-regulated in Landrace pigs, and more genes encoding proteins associated with cellular catabolism (12.50% versus 0% for T90 versus L90, *p *= 0.08166) and carbohydrate metabolism (12.50% versus 0% for T90 versus L90, *p *= 0.08166) were up-regulated in Tongcheng pigs (Figure 5c). We found that 48 unique tags had an abundance of at least 10 copies in one of the libraries and there was at least a 2.0-fold difference in expression (*p *< 0.01) between T90 and L90 (Additional data file 5). Within this group, genes related to muscle contraction were up-regulated in T90: *FKBP1A *(FK506 binding protein 1A, 12 kDa), *VDAC3 *(voltage-dependent anion channel 3), *TNNT1 *(troponin T type 1 (skeletal, slow)), *RTN4 *(reticulon 4), *TPM2 *(tropomyosin 2 (beta)), *MYH2 *(myosin, heavy polypeptide 2, skeletal muscle, adult), *ACTN2 *(actinin, alpha 2), *RYR1 *and *TNNT3 *(troponin T type 3). The expression of *SDHD *(succinate dehydrogenase complex, subunit D, integral membrane protein), *FMOD *(fibromodulin), *GNAS *and *CD63 *was higher in L90. Most conspicuously, the transcript for noncoding RNA, *TncRNA*, was also upregulated in T90. The transcript for *RPL29 *and a novel transcript corresponding to LongSAGE tag 'GGCGCAGGCGTGGGGGC', which fitted the criteria selected for both T33 versus L33 and T65 versus L65, were also up-regulated in L90.

### Longer cDNA sequences obtained from the novel SAGE tags

On average, 30% of the unique tags that we screened did not match any known sequence, particularly tags with lower copy numbers. These novel tags might, therefore, represent uncharacterized genes or transcripts. To convert novel tags into their corresponding cDNA fragments, the generation of longer cDNA fragments from serial analysis of gene expression tags for gene identification (GLGI) was carried out. A total of 113 longer cDNA sequences were experimentally obtained from 67 novel unique tags (Table 5). These ESTs ranged from 35-382 base-pairs (bp; mean 121 bp) in length. However, 100 sequences still matched no known sequence in the NCBI database. Six polyadenylation signals are frequently found in human transcripts [29]. Of these, 'AATAAA' and 'ATTAAA' had the highest frequencies among the unidentified genes (AATAAA, 50; ATTAAA, 24; AATAAT, 6; AATTA, 11; CATAAA, 5; AGTAAA, 5). Moreover, a total of 12 cDNA ends among these sequences contained two or three CATG sites, perhaps because of incomplete digestion at the 3'-most CATG consensus site by the anchor enzyme '*Nla*III'.

**Table 5:** cDNA sequence isolated by GLGI from novel LongSAGE tags

LongSAGE tag	Product size (bp)*	No. of UniGene matches	Abundance					
			
			T33	T65	T90	L33	L65	L90
CCCCATTGTACTTGAAC	116,81,92		10	0	8	5	5	6
CCCATTGTACTCGAACT	117,214		1	0	2	1	4	3
CCCATTGTACCTGAACT	134,402,116,151,73		3	0	2	0	1	3
CCCATTGTACTGGAACT	106,74		2	0	0	0	0	0
CCCATTGTACTTGAGCT	74,92,114		2	0	1	0	3	2
CCCATTGTACCTTGAAC	121		1	0	1	1	0	0
CCCATTGTACTTGACTT	115		1	0	1	0	1	1
CCCATTGTACTTGAACC	136,116		5	1	4	12	4	11
CCCATTGTACTTGGACT	102,128		3	0	3	0	8	2
CCCATTGTACTTGCACT	118		1	0	0	0	0	0
ACAGGAACTCCTTGCCT	104		1	0	0	0	0	0
TCTGGAGAAGTGGGGAG	77		5	0	1	0	0	0
CAGTTCTCCCACCTTAT	96		6	0	2	14	1	0
GGGCGTCTAAATGTGAA	89,115,121		1	14	1	1	1	0
TTCTTGATCTCTTCCTG	119,127		1	12	12	1	8	13
GCCACATCCTTTCTCCC	189,161,116,98,145		1	10	11	0	6	3
GACGAGATGGAGTTCAC	94,107,100		1	7	0	1	0	0
GATCGGGACATTGGGGC	87,81		1	8	5	1	6	3
GAGAAAACGAAGACAAG	136,65		2	12	12	0	6	5
GAAAATTGCCCCCCCCC	113,77		1	7	2	0	0	0
GACTAGCAATTTCGGTT	55,68		1	6	2	4	10	9
GGGCTGCTTTTTGTCAC	58,156		1	7	2	1	1	2
TGCTAGATTGGAGTGGG	93,124,150		1	6	1	2	8	4
AAGCTGTGGTTTGATCC	82		13	5	13	10	6	8
AGACAGACAGTTGCTGG	97,161		9	2	3	5	5	3
AGCATCCCAAACAAACA	79,116		27	13	46	15	26	49
ATGGGCCGTTAATAAAG	73		28	11	15	36	34	35
CAAACTCTTGCCCCGAT	73	1,458	7	1	3	1	1	0
CAGCAGGTGCTCAATAA	106	32,297	14	5	2	9	10	3
CCAACACTCATAGCAAT	71,161,100		16	6	16	22	16	27
CCCATTGGACTTGAACT	247		11	3	0	2	0	0
CCCATTGTACTTGAACT	134		448	130	577	501	448	515
GAACGCCTAATAAAGCA	106	32,126	9	2	5	7	1	8
CCTGACCCCACACGCCT	69		24	0	7	0	1	0
AGTAAACGGGCTGCTCC	134		10	0	2	0	0	0
TAGAGGTGCTGTCTATG	189,142		10	0	0	8	0	0
CTACTTTAGCACCTGCT	218		8	0	10	4	7	9
TCAAGCCTAGCAGTCTA	142		27	5	22	11	4	15
TGAATTTTGCATTCCAT	122	27,615	14	2	8	7	8	3
GTAGGGGAAGGAGGAGG	109,96		14	5	11	0	0	0
GGCTTCGGCTTGTTTGC	170,89		11	3	8	7	6	2
TGCCCTTTCCCCAAAGC	94		11	1	4	3	3	4
ATCTGCCGTTAATAAAG	89	947	207	81	211	167	220	219
CTGATTGGAACTGTATT	152	31,595	200	81	151	212	181	152
GGCTTCGGCTTGTTTGA	240	791	199	254	173	290	237	186
GCCCTGGGGCCTCAATA	92	37,210	111	158	96	118	123	75
GATTCCGTGAAGGAACA	73	19,414	54	56	67	78	68	57
AGGTTGCGGGTTCGGTC	113	23,946	54	65	35	83	86	62
GTTCGTGCCAAATTCCG	228	6,701	51	82	29	39	36	44
AAGATCAAGATTATTGC	159	10,316	22	47	36	9	41	20
CAGGGGCTTCAGTTGAT	364,239		0	0	0	29	23	32
ATTAAGAGGGACGGCCG	146		19	14	6	1	22	10
CACGCTTTCTTCAAAGC	302		3	5	13	24	9	34
GCGTGAATGTGAGCAGG	127,149		2	11	8	1	3	4
CGTGGGCAAAGCTGAAG	122,147		8	24	11	14	22	10
CTCGTTCTGAAATAAAG	68,168,186		1	19	76	1	37	70
AGGATGCCGGTACGATC	189,170,107		7	23	7	14	18	7
TCTCAGAATTAGCTTTG	89	22,100	5	20	17	3	14	20
GTTTTGCTGCTTCCCAA	132		2	18	9	0	8	15
CCTGCCCTGCCCTATTC	77,68		4	16	0	4	3	0
AGCTATGGCTTAGGCCA	161,126		1	11	3	0	1	0
TTTACTCAACCTTTGGT	96,169	13,955	3	28	37	1	21	21
TGGGCAGCCTTCCCTTC	221		1	11	4	0	2	1
CCAGAAGTAAGGCTTTC	123		5	16	10	0	0	0
AATCCAGGATGCGGCTG	70,173		1	9	0	0	4	3
GCATCTAGCTCCTCATT	151		1	16	8	1	2	2
TCGGACGTACATCGTTA	145		4	13	10	14	9	7

All cDNA sequences obtained from GLGI analysis were deposited in the NCBI database (Additional data file 8). *Multiple values indicate that more than one sequence was obtained from a tag in the GLGI experiment, and these sequences were of different lengths.

## Discussion

To our knowledge, the present study is the first full-transcriptome analysis of skeletal muscle from porcine fetuses of Tongcheng and Landrace pigs at different stages (33, 65 and 90 dpc). In the clones that we identified in our LongSAGE libraries, the GC content was about 44.56% to 50.02%, indicating that AT-rich tags were retained during library construction [30] and that our experiments produced no inherent GC bias [31]. Among the 14,683 unique tags that we analyzed further, 225 (1.53%) matched more than one UniGene sequence. Hence, the LongSAGE unique tags are also more representative of the corresponding gene information. In addition, the differential expression patterns of 12 selected genes at the mRNA level identified by QPCR and LongSAGE (r = 0.79, p = 8.52E-17) agreed well, suggesting that our LongSAGE data can be reliably utilized for a comprehensive study of gene expression profiles in skeletal muscle. Unfortunately, however, many of our LongSAGE tags did not match any of the currently known sequences in pig. This limitation in the cDNA resources that have been deposited for this animal restricted the amount of useful mining information obtainable from our LongSAGE data. At the same time, this indicates that many porcine genes have yet to be identified. Chen *et al*. [32] reported, using the GLGI method, that about 70% of the unmatched SAGE tags in human were derived from novel transcripts. Our GLGI experiment also suggested that most of the novel tags had come from unknown transcripts. The combined GLGI/LongSAGE approach therefore has the potential to provide an effective strategy for identifying novel genes and transcripts in the pig.

We first analyzed such differences in prenatal skeletal muscle development between indigenous and exotic breed pigs on the basis of gene expression profiling using LongSAGE. Differences in the developmental features of Landrace and Tongcheng pigs were indicated by transcriptome clustering and gene expression patterns during skeletal muscle development. The transcription profiles at 65 and 90 dpc were more similar in Landrace than Tongcheng pigs. Analysis of biological function suggested that the LongSAGE tag clusters differed significantly between the two breeds in certain functional categories of genes and expression patterns. Muscle development, mitochondrial and ribosomal proteins were enriched in both Tongcheng and Landrace pigs, but the genes in these functional categories exhibited different expression patterns in the two breeds. These results indicate differences between Tongcheng and Landrace pigs in the synchronization of events during skeletal muscle development, and show that skeletal muscle grows more rapidly in Landrace pigs at the stages selected. Differences in embryo growth between indigenous Chinese and western breeds have been observed as early as 12 dpc [10-12]. The lack of synchronicity of skeletal muscle development between these two breeds will need to be further investigated in future studies.

Primary myotube formation occurs at 35 dpc in the pig. Our results show that genes encoding proteins involved in muscle fiber construction and contraction were up-regulated in the T33 samples, but some growth factors that promote myoblast differentiation, such as *IGF2 *and *MDK*, were significantly more abundant in L33 than in T33. IGF2 is an autocrine survival factor for differentiating myoblasts [33]. The regulatory mutation is important for increasing meat production, and its expression levels have been shown to differ between obese and lean genotypes in postnatal pigs [34]. However, the differences between genotypes in *IGF2 *mRNA expression in embryonic skeletal muscle remain poorly understood. In the present study, muscle *IGF2 *expression was observed to increase to a peak at 90 dpc in both breeds. Also, *IGF2 *was more highly expressed in Landrace than Tongcheng pigs at both 33 dpc and 65 dpc, but no significant differences between the breeds were found for this gene at 90 dpc. Midkine, a heparin-binding growth factor, is expressed in both proliferating and differentiated cells, but is more highly expressed in less differentiated cells [35]. We found that *MDK *was decreased in both Tongcheng and Landrace pigs as myogenesis progressed, which is consistent with previous studies [36]. Comparison of the two breeds at the same gestational stages further revealed that *MDK *expression was higher in L33 (*p *< 0.01), and decreased more rapidly in Landrace pigs with the onset of myogenesis.

The expression levels of *PMTA*, *GSTP1 *and *CRABP1*, which are associated with the anti-apoptotic pathway, were significantly higher in L33 than T33. PTMA, which is localized in the mitotic spindle during mitosis, plays a role in cell proliferation and anti-apoptosis [37,38]. *MARCKS*, which is involved in myoblast fusion, was also more highly expressed in L33. Calpain-mediated proteolysis of phosphorylated MARCKS is a prerequisite for myoblast fusion, but over-expression of *MARCKS *significantly abrogates the fusion process [39]. In contrast, *CAPNS1*, which is associated with the endoplasmic reticulum (ER) stress-induced apoptotic response, was more highly expressed in T33 than L33. Furthermore, caspase 3, apoptosis-related cysteine peptidase (*CASP3*), an ER stress-specific caspase, was detectable in T33 but not in L33 (3 versus 0 for T33 versus L33 in expression abundance). Proliferating myoblasts are far more susceptible to apoptotic cell death than terminally differentiated myotubes [40]. Nakanishi *et al*. [41] reported that about 15% of C2C12 cells die during the first 24 hours of incubation in differentiation medium. This phenomenon, induced by ER stress factors, has also been detected *in vivo *[41]. Hence, the survival of myoblasts is important for controlling the deposition of muscle mass during embryonic development [40] and this is regulated by growth factors and anti-apoptotic factors. In this regard, our current data show that *IGF2 *and *MDK *are important for maintaining the survival of myoblasts and also indicate that myoblast growth status differs between the Tongcheng and Landrace breeds at 33 dpc.

Primary muscle fiber formation ceases and secondary muscle fibers are assembled in pigs at 65 dpc. The myoblasts are terminally differentiated and the shape of the myofibers is very clear at this stage [13]. But electron microscopy indicated differences in sarcomere length and myofilament thickness between the two breeds (data not shown). As myoblasts cease to proliferate, the continuing development of muscle involves growth without cell division [42]. Cell growth requires increased protein synthesis, which can be assayed by ribosome synthesis [43]; about 50% of nuclear transcription is associated with ribosome synthesis in growing mammalian cells [44]. In our current SAGE libraries, we detected 59 genes that encode ribosome proteins, accounting for 7.6% (24,135/317,115) of the total number of LongSAGE tags. Of these ribosome protein transcripts, 39 were significantly different between the two pig breeds at 65 dpc. Among these, 17 were more highly expressed in Tongcheng pigs and 22 in the Landrace variety. However, there were far more transcripts with ≥2.0-fold differences in expression between T65 and L65 in Landrace than in Tongcheng pigs (15/5). Elongation factors were also more highly expressed in L65 than T65.

*TTN *was up-regulated in L65, while *FHL1C *and *YWHAQ *were under-expressed in L65 compared with T65. *TTN *not only encodes a protein that forms part of the muscle fibers but also acts as a signaling complex, promoting skeletal muscle development [45]. *FHL1C *is an alternatively spliced isoform of *FHL1*, with a specific expression profile in testis, skeletal muscle and heart that differs from the more widely expressed *FHL1 *gene [46]. YWHAQ is the theta isomer of the 14-3-3 family of proteins that function as both cell cycle- and apoptosis-related regulators [47]. Interestingly, *GNB2L1 *and *TPT1*, which are involved in regulating translation, were also up-regulated in L65. *GNB2L1*, a member of the receptor family for activated C-kinase 1, has a role in the regulation of cell cycle arrest, cell movement and cell growth [48]. Over-expression or down-regulation of this gene can result in reduced cell growth [49]. Also, ribosome activation is regulated by *GNB2L1 *via the integrin beta-*GNB2L1*-PKC complex [48,50]. This gene was highly expressed in both Landrace and Tongcheng pigs at 33 dpc (128 versus 137 for L33 versus T33 in expression abundance) and 90 dpc (109 versus 140 for L90 versus T90 in expression abundance), but its expression was significantly higher in L65 than T65 (140 versus 41 for L65 versus T65 in expression abundance). On the other hand, integrin beta 1 (*ITGB1*), a member of the integrin beta family, was also up-regulated in L65. *TPT1 *encodes a ubiquitously expressed protein that plays a role in the cell growth and anti-apoptotic pathways. It regulates the efficiency of protein synthesis by stabilizing the GDP form of EEF1A [51]. *TPT1 *was highly expressed in all six libraries, but significant differences were detected between the two pig breeds at 65 dpc (220 versus 101 for L65 versus T65 in expression abundance, p < 0.01). These results suggest that the growth rate of muscle cells was more rapid in Landrace than in Tongcheng pigs at 65 dpc.

The myosin heavy chain genes comprise *MYH3*, *MYH8 *(myosin, heavy chain 8, skeletal muscle, perinatal), *MYH2*, *MYH1 *(myosin, heavy chain 1, skeletal muscle, adult), and *MYH4 *(myosin, heavy chain 4, skeletal muscle). The *MYH3 *and *MYH8 *isoforms are expressed during development and the other three genes are expressed in trunk skeletal muscle [52]. In the present study, expression of *MYH3 *and *MYL4 *peaked at 65 dpc, whereas *MYH2 *was undetectable at 33 dpc and maximally expressed at 90 dpc. Genes encoding proteins involved in muscle fiber contraction were also up-regulated in T90 samples: *TNNT1*, *TPM2*, *MYH2*, *ACTN2*, *RYR1 *and *TNNT3*. In contrast, genes involved in signal transduction were up-regulated in L90: *SYNJ2BP *(synaptojanin 2 binding protein) and *FMOD*. SYNJ2BP, also termed Arip2, is a factor regulating activin A receptor type IIA (ACVR2A) expression and activin function, which plays an important role in the transforming growth factor (TGF)β signal pathway [53]. *FMOD *encodes a member of a family of small interstitial proteoglycans that regulate TGFβ activity by sequestering it in the extracellular matrix [54]. Intriguingly, we found that one differentially expressed tag represented a noncoding RNA and showed homology to human *TncRNA*, a trophoblast-derived noncoding RNA. The expression of this product increased with the progression of myogenesis in both pig breeds and significant differences could be detected at only 90 dpc (60 versus 18 for T90 versus L90 in expression abundance). Recently, Timmons *et al*. [55] reported that *TncRNA *is down-regulated in Duchenne muscular dystrophy but is up-regulated during exercise. Geirsson *et al*. [56] also reported that *TncRNA *inhibits class II major histocompatibility complex transactivator-mediated transcription. These findings suggest that noncoding RNA species could well be functional during muscle formation.

## Conclusion

The present study provides a rich new information resource that increases our understanding of the molecular mechanisms underlying porcine skeletal muscle development via comparative analyses of indigenous Chinese and exotic breeds. Our comparative analysis of the prenatal skeletal muscle transcriptomes of obese and lean type pig breeds suggests that skeletal muscle grows more slowly and undergoes more complicated changes in molecular events in Tongcheng than in Landrace pigs at the stages selected. This finding could contribute to explaining the superior perceived meat quality of Tongcheng pigs. The cellular functions of the differentially expressed transcripts that matched annotated genes revealed that each stage in development showed characteristic differences between the two breeds in various functional categories: muscle development, apoptosis, protein synthesis, signaling transduction, and so on. The up-regulation of genes associated with increased cellular growth and myoblast survival in Landrace pigs was responsible for faster muscle growth. More generally, our data are likely to be helpful in uncovering the pathways that mediate prenatal skeletal muscle development in vertebrates. A number of differentially expressed genes were identified between stages and breeds, including candidate genes associated with meat production traits, which may be commercially valuable. In addition, several thousand novel tags derived from unknown genes were screened, indicating that many porcine genes remain to be characterized. Our combined GLGI/LongSAGE method also provides a new strategy for annotating the porcine genome. Finally, our data are also likely to help in identifying genes underlying some human diseases. However, although most biological activities are carried out by proteins, we have focused only on mRNA expression levels in prenatal skeletal muscle. Therefore, details about protein levels would be more helpful for understanding these issues.

## Materials and methods

### Animals and tissue preparation

All animal procedures were performed according to protocols approved by Hubei Province, PR China for Biological Studies Animal Care and Use Committee. Tongcheng and Swedish Landrace sows (15 sows for each breed) were mated with the boar of the corresponding breed. The sows were then sacrificed at a commercial slaughterhouse at 33, 65 and 90 dpc (five sows at each stage for each breed). The uteri containing the fetuses were collected immediately, and the longissimus muscle tissues were rapidly and manually dissected from each fetus. These samples were snap-frozen in liquid nitrogen and stored at -80°C until further use. Four fetuses (two males and two females) from one sow were used for constructing each LongSAGE library. Subsequently, skeletal muscles from 72 fetuses were used for QPCR validation.

### RNA extraction and LongSAGE library construction

Total RNA was prepared from the frozen longissimus muscle using TRIZOL Reagent^® ^(Invitrogen, California, USA) and digested by RNase-free DNase I. The quality of the RNA was evaluated by spectrophotometry and agarose gel electrophoresis.

For the skeletal muscles from the six different samples, T33, T65 and T90 from Tongcheng pigs and L33, L65 and L90 from Landrace pigs, equal quantities of total RNA from four individuals (*n *= 4) obtained from one sow were pooled. About 30 μg purified total RNA was used for the construction of each library. Six LongSAGE libraries were generated using I-SAGE™ Long kits (Invitrogen) according to the manufacturer's instructions. Transforming clones were sequenced with the help of an ABI PRIZM 3730 DNA sequencer. Phred software was used to determine the confidence of base calling; sequences with Phred score >20 were considered reliable [57,58].

### SAGE data analysis

The SAGE 2000 software version 4.5 (Invitrogen) was used to extract LongSAGE tags and eliminate duplicate ditags. All unique tags that were observed no less than twice in at least one library were selected for further comparison. Differential expression was determined by analyzing the significance of tag frequency differences between any of the LongSAGE libraries using chi-square analysis and Monte-Carlo simulation [59]. A *P *value <0.05 was considered significant. A reference database (SAGEmap_tag_ug-rel.zip for *Sus scrofa*) was downloaded from the National Center for Biotechnology Information (NCBI) [60] to identify the genes represented by the LongSAGE tags (17 bp).

### Quantitative PCR

First-strand cDNA was synthesized using a RevertAid™ First Strand cDNA Synthesis kit (MBI Fermentas, Vilnius, Lithuania) and oligo(dT) with 4 μg RNA, and subsequently diluted with nuclease-free water (Sigma, Saint Louis Mo, USA) to 12.5 ng/μl cDNA. Twelve differentially expressed genes (*MYLPF*, *MYL2*, *MYL1*, *SLN*, *TNNC2*, *TOB1*, *CRABP1*, *LGALS1*, *GNB2L1*, *TPT1*, *RPS28 *and *TncRNA*) identified in the SAGE experiment were selected and analyzed by QPCR. Histone mRNA (*H3F3A*), which was consistently expressed in all LongSAGE libraries, was used as an internal control for normalization purposes. Each QPCR reaction (in 20 μl) contained 1 × PCR buffer (TaKaRa, Dalian, China), 3.0 mM MgCl_2_, 100 μM of each dNTP, 0.3 μM primers (Table 6), 0.3 × SYBR Green I, 2 U Taq DNA polymerase (TaKaRa) and 2 μl of normalized template cDNA. The cycling conditions consisted of an initial, single cycle of 30 s at 95°C followed by 45 cycles of 5 s at 95°C, 15 s at annealing temperature (Table 6) and 20 s at 72°C. All PCR amplifications were performed in triplicate for each RNA sample and gene expression levels were quantified relative to *H3F3A *expression using Gene Expression Macro software (Bio-Rad, Richmond, CA, USA). The results were analyzed using the 2^-ΔΔCt ^method described previously [61]. Data are presented as fold changes in gene expression normalized to the *H3F3A *gene and relative to the T33 sample. For the T33 sample, ΔΔCt equaled zero and 2^0 ^equals one, so that the fold change in gene expression relative to the T33 sample equals one, by definition. For the other samples, evaluation of 2^-ΔΔCt ^indicated the fold change in gene expression relative to the T33 sample. Dissociation curves were generated to ensure that a single amplicon had been produced. Differences in gene expression between groups were evaluated using Student's *t*-test and were considered statistically significant at *p *< 0.05.

**Table 6:** Primer sequences and PCR product sizes of genes selected for validation by QPCR

Gene	GenBank ID	Primer sequence	Annealing Tm (°C)	Product size (bp)
*H3F3A*	NM_213930.1	Forward 5'-GCAAGAGTGCGCCCTCTACT-3' Reverse 5'-TTGGCATAATTGTTACACGTTTGG-3'	60	288
*TNNC2*	NM_001001862.1	Forward 5'-AAGGAGTTGGGCACCGTGAT-3' Reverse 5'-CGGCCTTCGTTGTTCTTGTC-3'	60	326
*MYL2*	NM_213791.1	Forward 5'-GGGTGCTCAGGGCTGATTAT-3' Reverse 5'-AGGCTGCAAAGAAGATGAAGGT-3'	60	326
*MYL1*	NM_214374.1	Forward 5'-GACTTTGTTGAGGGTCTGCG-3' Reverse 5'-GAGTGGTGCTTGGATTTGAG-3'	60	452
*SLN*	Z98820.2	Forward 5'-AGAATGGAGCGATCCACCCG-3' Reverse 5'-AAACACTTGGCAGCCCTTGA-3'	60	300
*MYLPF*	NM_001006592.1	Forward 5'-GAGAAGGGCAGCGGCAGAAG-3' Reverse 5'-GTGCGTGATGACGTAGCAGATGTT-3'	60	466
*TOB1*	EF486515	Forward 5'-TTACCACTGCCACTTTCGCT-3' Reverse 5'-TTCTGCTTCAAGAGGTCATTCAC-3'	61	129
*CRABP1*	EF397416	Forward 5'-GTGTGAACGCCATGCTGAG-3' Reverse 5'-CGTCCGTCCACTGTCTCC-3'	55	169
*LGALS1*	AY604429	Forward 5'-GGTCGCCAGCAACCTGAATCTC-3' Reverse 5'-GTCTCCGTGCATGTCGAAGCG-3'	58	151
*GNB2L1*	NM_214332	Forward 5'-GCTGGGACAAGCTGGTCAAGG-3' Reverse 5'-AGCACAGAGCCAGTAGCGATTG-3'	58	245
*TPT1*	NM_214373	Forward 5'-GGCTGTTGGGATCGGATCTATC-3' Reverse 5'-AACAATGCCTCCGCTCCAAAG-3'	55	150
*RPS28*	NM_001001587	Forward 5'-GGCAGGACAGGTTCGCAGG-3' Reverse 5'-ATATCCAGGACCCAGCCACAAC-3'	56	179
*TncRNA*	EF397601	Forward 5'-GACCGCTGTCGTCACTGTATG-3' Reverse 5'-AGCACTTGCCCAGCCCTAG-3'	55	189

### Cluster analysis

To characterize the gene expression profiles in selected longissimus muscle samples further, an expression profile cluster analysis was performed utilizing Cluster 3.0 and TreeView software [62]. The normalization process included logarithmic transformation of the data, which was carried out as described by Nacht *et al*. [63]. A hypothetical tree-like diagram, which describes 'evolutionary' relationships between different datasets, was constructed using the TreeBuild 3D viewer with all the tags represented in our SAGE libraries. In addition, SAGE Data Analysis 2.0 software developed by Cai *et al*. [64] was used to identify differentially expressed genes that behaved similarly throughout skeletal muscle development in both pig breeds.

### Gene Ontology annotation

To link tag identity with putative gene function, UniGene clusters of reliably annotated tags, which were significantly differentially expressed during development in each pig breed, were retrieved using GO annotation for the category 'biological process' [65]. For known genes in each catalog, the number of occurrences of a GO term in any given GO category (biological process) was searched using the Blast2GO program that was used for GO annotation [66]. On the basis of the differentially expressed genes, the functional catalogs in different muscles were compared using FatiGO software with reference to the functions of these genes in human [67]. *P *values <0.05 were considered significant, and 0.05 <*p *< 0.1 indicated a tendency. Expression Analysis Systematic Explorer (EASE) software was used for functional analysis of genes over-represented in the expression pattern cluster [68]. An EASE score (Jackknife one-sided Fisher exact *p *values) <0.05 was considered significant.

### Generation of longer cDNA fragments from serial analysis of gene expression tags for gene identification

To analyze novel LongSAGE tags further, GLGI was carried out using the 3' cDNA sample that had been used previously for LongSAGE analysis [32]. GLGI amplification, with slight modifications, was then performed for each tag. The sense primers (5'-CATGxxxxxxxxxxxxxxxxx-3', where x represents a 17 bp sequence of the tag), were designed on the basis of each LongSAGE tag instead of the sense primers (5'-GGATCCCATGxxxxxxxxxx-3', where x represents a 10 bp sequence of the tag from the original SAGE), as in the original GLGI. The anti-sense primer used was 5'-ACTATCTAGAGCGGCCGCTT-3', which corresponds to the 3' end of all of the cDNAs generated by GLGI reverse transcription primers. The PCR conditions and amplified products were then treated as previously described by Chen *et al*. [32]. All the sequences generated from the clones were subjected to a basic local alignment search tool (BLAST) search. Those containing the LongSAGE tags did not match any known sequence with more than 85% homology in the same orientation, and were defined as genuine novel sequences.

## Additional data files

The following additional data are available with the online version of this paper. Additional data file 1 is a table listing longSAGE tags expressed differentially in Tongcheng pigs. Additional data file 2 is a table listing longSAGE tags expressed differentially in Landrace pigs. Additional data file 3 is a table listing genes expressed differentially between breeds at 33 dpc. Additional data file 4 is a table listing genes expressed differentially between breeds at 65 dpc. Additional data file 5 is a table listing genes expressed differentially between breeds at 90 dpc. Additional data file 6 provides cluster-analysis results of differentially expressed LongSAGE tags separated by breed. Cluster analysis was based on 1,400 and 1,201 transcripts differentially expressed during skeletal muscle development in Tongcheng and Landrace pigs, respectively. SAGE libraries are plotted on the x-axis, and tag abundance, plotted as a fraction of the total tags for a gene in the library in question, is shown on the y-axis. T = Tongcheng; L = Landrace; numbers 33, 65, and 90 indicate days post coitus. Eight clusters for Tongcheng pig are shown in (A1-A8). Landrace clusters are shown in (B1-B8). Additional data file 7 lists the references for the genes listed in Table 4. Additional data file 8 lists the GenBank accession numbers of the cDNA sequences obtained from GLGI experiments.

## Supplementary Material

Additional data file 1 LongSAGE tags expressed differentially in Tongcheng pigs. Click here for file

Additional data file 2 LongSAGE tags expressed differentially in Landrace pigs. Click here for file

Additional data file 3 Genes expressed differentially between breeds at 33 dpc. Click here for file

Additional data file 4 Genes expressed differentially between breeds at 65 dpc. Click here for file

Additional data file 5 Genes expressed differentially between breeds at 90 dpc. Click here for file

Additional data file 6 Cluster analysis was based on 1,400 and 1,201 transcripts differentially expressed during skeletal muscle development in Tongcheng and Landrace pigs, respectively. SAGE libraries are plotted on the x-axis, and tag abundance, plotted as a fraction of the total tags for a gene in the library in question, is shown on the y-axis. T = Tongcheng; L = Landrace; numbers 33, 65, and 90 indicate days post coitus. Eight clusters for Tongcheng pig are shown in (A1-A8). Landrace clusters are shown in (B1-B8). Click here for file

Additional data file 7 References for the genes listed in Table 4. Click here for file

Additional data file 8 GenBank accession numbers of the cDNA sequences obtained from GLGI experiments. Click here for file
