# Supplementary material for: LongSAGE analysis of skeletal muscle at three prenatal stages in Tongcheng and Landrace pigs
Source: Genome Biol. 2007 Jun 16;8(6):R115. doi: 10.1186/gb-2007-8-6-r115 (PMC2394763; doi:10.1186/gb-2007-8-6-r115)
Supplement: Additional data file 7 — References for the genes listed in Table 4. [file gb-2007-8-6-r115-S7.doc]

**Supplementary References**

This supplementary data describe the references of genes listed in table 4 of the text.

1. Liao W, Hong SH, Chan BH, Rudolph FB, Clark SC, Chan L: **APOBEC-2, a cardiac- and skeletal muscle-specific member of the cytidine deaminase supergene family.** *Biochem Biophys Res Commun* 1999, **260**: 398-404.
2. Wechsler-Reya RJ, Elliott KJ, Prendergast GC: **A role for the putative tumor suppressor Bin1 in muscle cell differentiation.** *Mol Cell Biol* 1998, **18**: 566-575.
3. Pisani DF, Cabane C, Derijard B, Dechesne CA: **The topoisomerase 1-interacting protein BTBD1 is essential for muscle cell differentiation.** *Cell Death Differ* 2004, **11**: 1157-1165.
4. Leuranguer V, Papadopoulos S, Beam KG: **Organization of calcium channel beta1a subunits in triad junctions in skeletal muscle.** *J Biol Chem* 2006, **281**: 3521-3527.
5. Kelly RG, Zammit PS, Schneider A, Alonso S, Biben C, Buckingham ME: **Embryonic and fetal myogenic programs act through separate enhancers at the MLC1F/3F locus.** *Dev Biol* 1997, **187**: 183-199.
6. Takada F, Vander Woude DL, Tong HQ, Thompson TG, Watkins SC, Kunkel LM, Beggs AH: **Myozenin: an alpha-actinin- and gamma-filamin-binding protein of skeletal muscle Z lines.** *Proc Natl Acad Sci U S A* 2001, **98**: 1595-1600.
7. Suzuki J, Kaziro Y, Koide H: **Positive regulation of skeletal myogenesis by R-Ras.** *Oncogene* 2000, **19**: 1138-1146.
8. Magnusson C, Libelius R, Tagerud S: **Nogo (Reticulon 4) expression in innervated and denervated mouse skeletal muscle.** *Mol Cell Neurosci* 2003, 22: 298-307.
9. Scartezzini P, Egeo A, Colella S, Fumagalli P, Arrigo P, Nizetic D, Taramelli R, Rasore-Quartino A: **Cloning a new human gene from chromosome 21q22.3 encoding a glutamic acid-rich protein expressed in heart and skeletal muscle.** *Hum Genet* 1997, **99**: 387-392.
10. Vangheluwe P, Schuermans M, Zador E, Waelkens E, Raeymaekers L, Wuytack F: **Sarcolipin and phospholamban mRNA and protein expression in cardiac and skeletal muscle of different species.** *Biochem J* 2005, **389**: 151-159.
11. Nicholas G, Thomas M, Langley B, Somers W, Patel K, Kemp CF, Sharma M, Kambadur R: **Titin-cap associates with, and regulates secretion of, Myostatin.** *J Cell Physiol* 2002, **193**:120-131.
12. Almenar-Queralt A, Gregorio CC, Fowler VM: **Tropomodulin assembles early in myofibrillogenesis in chick skeletal muscle: evidence that thin filaments rearrange to form striated myofibrils.** *J Cell Sci* 1999, **112**: 1111-1123.
13. Gahlmann R, Kedes L: **Cloning, structural analysis, and expression of the human fast twitch skeletal muscle troponin C gene.** *J Biol Chem* 1990, 265: 12520-12528.
14. Tiso N, Majetti M, Stanchi F, Rampazzo A, Zimbello R, Nava A, Danieli GA: **Fine mapping and genomic structure of ACTN2, the human gene coding for the sarcomeric isoform of alpha-actinin-2, expressed in skeletal and cardiac muscle.** *Biochem Biophys Res Commun* 1999, **265**: 256-259.
15. Morisaki T, Holmes EW: **Functionally distinct elements are required for expression of the AMPD1 gene in myocytes.** *Mol Cell Biol* 1993, **13**: 5854-5860.
16. Odermatt A, Taschner PE, Khanna VK, Busch HF, Karpati G, Jablecki CK, Breuning MH, MacLennan DH: **Mutations in the gene-encoding SERCA1, the fast-twitch skeletal muscle sarcoplasmic reticulum Ca2+ ATPase, are associated with Brody disease.** *Nature Genetics* 1996, **14**: 191-194.
17. Feo S, Antona V, Barbieri G, Passantino R, Cali L, Giallongo A: **Transcription of the human beta enolase gene (ENO-3) is regulated by an intronic muscle-specific enhancer that binds myocyte-specific enhancer factor 2 proteins and ubiquitous G-rich-box binding factors.** *Mol Cell Biol* 1995, **15**: 5991-6002.
18. Almenar-Queralt A, Lee A, Conley CA, Ribas de Pouplana L, Fowler VM: **Identification of a novel tropomodulin isoform, skeletal tropomodulin, that caps actin filament pointed ends in fast skeletal muscle.** *J Biol Chem* 1999, **274**: 28466-28475.
19. Hittel DS, Hathout Y, Hoffman EP, Houmard JA: **Proteome analysis of skeletal muscle from obese and morbidly obese women.** *Diabetes* 2005, **54**: 1283-1288
20. Wilson EM, Hsieh MM, Rotwein P: **Autocrine growth factor signaling by insulin-like growth factor-II mediates MyoD-stimulated myocyte maturation.** *J Biol Chem* 2003, **278**: 41109-411013.
21. Cai XF, Tao Z, Yan ZQ, Yang SL, Gong Y: **Molecular cloning, characterisation and tissue-specific expression of human LAG3, a member of the novel Lag1 protein family.** *DNA Seq* 2003, **14**: 79-86.
22. Motamed K, Blake DJ, Angello JC, Allen BL, Rapraeger AC, Hauschka SD, Sage EH: **Fibroblast growth factor receptor-1 mediates the inhibition of endothelial cell proliferation and the promotion of skeletal myoblast differentiation by SPARC: a role for protein kinase A.** *J Cell Biochem* 2003, **90**: 408-423.
23. Verschuure P, Tatard C, Boelens WC, Grongnet JF, David JC: **Expression of small heat shock proteins HspB2, HspB8, Hsp20 and cvHsp in different tissues of the perinatal developing pig**. *Eur J Cell Biol*. 2003, **82**: 5523-30
24. De Angelis R, Iezzi S, Bruno T, Corbi N, Di Padova M, Floridi A, Fanciulli M, Passananti C: **Functional interaction of the subunit 3 of RNA polymerase II (RPB3) with transcription factor-4 (ATF4).** *FEBS Lett*. 2003, **547**: 15-19.
25. Faerman A, Shani M: **The expression of the regulatory myosin light chain 2 gene during mouse embryogenesis.** *Development* 1993, **118**: 919-929.
26. Arcuri C, Giambanco I, Bianchi R, Donato R: **Subcellular localization of S100A11 (S100C, calgizzarin) in developing and adult avian skeletal muscles.** *Biochim Biophys Acta* 2002, **1600**: 84-94.
27. Park EC, Shim S, Han JK: **Identification and expression of XRTN2 and XRTN3 during Xenopus development.** *Dev Dyn* 2005, **233**: 240-247.
28. Lawler J, Duquette M, Whittaker CA, Adams JC, McHenry K, DeSimone DW: **Identification and characterization of thrombospondin-4, a new member of the thrombospondin gene family.** *J Cell Biol* 1993, **120**: 1059-1067.
29. Ogata T, Oishi Y, Roy RR, Ohmori H: **Endogenous expression and developmental changes of HSP72 in rat skeletal muscles.** *J Appl Physiol* 2003, **95**: 1279-1286.
30. Krempler A, Kollers S, Fries R, Brenig B: **Isolation and characterization of a new FHL1 variant (FHL1C) from porcine skeletal muscle.** *Cytogenet Cell Genet* 2000, **90**: 106-114.
31. McKinsey TA, Zhang CL, Lu J, Olson EN: **Signal-dependent nuclear export of a histone deacetylase regulates muscle differentiation.** *Nature* 2000 **408**: 106-111.
32. Zhang CL, McKinsey TA, Olson EN: **The transcriptional corepressor MITR is a signal-responsive inhibitor of myogenesis.** *Proc Natl Acad Sci U S A* 2001, **98**: 7354-7359.
33. Evangelisti C, Riccio M, Faenza I, Zini N, Hozumi Y, Goto K, Cocco L, Martelli AM: **Subnuclear localization and differentiation-dependent increased expression of DGK-zeta in C2C12 mouse myoblasts.** *J Cell Physiol* 2006, **209**: 370-378.
34. Kwon HJ, Bae S, Son YH, Chung HM: **Expression of the Xenopus homologue of the receptor for activated C-kinase 1 (RACK1) in the Xenopus embryo.** Dev Genes Evol 2001, **211**: 195-197.
35. Ono S, Minami N, Abe H, Obinata T: **Characterization of a novel cofilin isoform that is predominantly expressed in mammalian skeletal muscle.** *J Biol Chem* 1994, **269**: 15280-15286.
36. Wei L, Zhou W, Wang L, Schwartz RJ: **beta (1)-integrin and PI 3-kinase regulate RhoA-dependent activation of skeletal alpha-actin promoter in myoblasts.** *Am J Physiol Heart Circ Physiol* 2000, **278**: H1736-1743.
37. Belkin AM, Burridge K: **Expression and localization of the phosphoglucomutase-related cytoskeletal protein, aciculin, in skeletal muscle.** *J Cell Sci* 1994, **107**: 1993-2003.
38. Mori K, Maeda Y, Kitaura H, Taira T, Iguchi-Ariga SM, Ariga H. **MM-1, a novel c-Myc-associating protein that represses transcriptional activity of c-Myc.** *J Biol Chem* 1998, **273**: 29794-29800.
39. Goldring K, Jones GE, Thiagarajah R, Watt DJ: **The effect of galectin-1 on the differentiation of fibroblasts and myoblasts in vitro**. *J Cell Sci* 2002, **115**: 355-366.
40. Scholz A, Hinssen H: **Biphasic pattern of gelsolin expression and variations in gelsolin-actin interactions during myogenesis.** *Exp Cell Res* 1995, **219**: 384-391.
41. O'Brien SP, Seipel K, Medley QG, Bronson R, Segal R, Streuli M: **Skeletal muscle deformity and neuronal disorder in Trio exchange factor-deficient mouse embryos.** *Proc Natl Acad Sci U S A* 2000, **97**: 12074-12078.
42. Yotov WV, St-Arnaud R: **Differential splicing-in of a proline-rich exon converts alphaNAC into a muscle-specific transcription factor.** *Genes Dev* 1996, **10**: 1763-1772.
43. Gregoire S, Yang XJ. **Association with class IIa histone deacetylases upregulates the sumoylation of MEF2 transcription factors**. *Mol Cell Biol* 2005, **25**: 2273-2287.
44. Wang HL, Wang H, Zhu ZM, Wang CF, Zhu MJ, Mo de L, Yang SL, Li K: **Subcellular localization, expression patterns, SNPs and association analyses of the porcine HUMMLC2B gene.** *Mol Genet Genomics* 2006, **276**: 264-272.
45. Zhu ZM, Zhang JB, Li K, Zhao SH: **Cloning, mapping and association study with carcass traits of the porcine SDHD gene.** *Anim Genet* 2005, **36**: 191-195.
46. Goldfarb LG, Park KY, Cervenakova L, Gorokhova S, Lee HS, Vasconcelos O, Nagle JW, Semino-Mora C, Sivakumar K, Dalakas MC: **Missense mutations in desmin associated with familial cardiac and skeletal myopathy.** *Nat Genet* 1998, **19**: 402-403.
47. Kyselovic J, Leddy JJ, Ray A, Wigle J, Tuana BS: **Temporal differences in the induction of dihydropyridine receptor subunits and ryanodine receptors during skeletal muscle development.** J Biol Chem. 1994, **269**: 21770-21777.
48. Schweighoffer F, Maire P, Tuil D, Gautron S, Daegelen D, Bachner L, Kahn A: **In vivo developmental modifications of the expression of genes encoding muscle-specific enzymes in rat.** *J Biol Chem* 1986, **261**: 10271-10276.
49. Schiaffino S, Reggiani C: **Molecular diversity of myofibrillar proteins: gene regulation and functional significance.** *Physiol Rev* 1996, **76**: 371-423.
50. <http://www.genecards.org/index.shtml>.

**Note**:

1. Genes encoding myofibrillar proteins including *MYBPC1*, *MYH2*, *MYH3*, *TPM1*, *TPM2*, *TPM3*, *TPM4*, *TNNC1*, *TNNI1*, *TNNT1*, and *TNNT3* were reviewed from [49].

2. Other genes including *CCNG1*, *FAU*, *PSMD2*, *SERF1A*, and *TIMM13*which were listed in table 4 of the text were detected to be expressed highly in skeletal muscle through searching the GeneCards database (<http://www.genecards.org/index.shtml>).
